# The Cell Ontology 2016: enhanced content, modularization, and ontology interoperability

**DOI:** 10.1186/s13326-016-0088-7

**Published:** 2016-07-04

**Authors:** Alexander D. Diehl, Terrence F. Meehan, Yvonne M. Bradford, Matthew H. Brush, Wasila M. Dahdul, David S. Dougall, Yongqun He, David Osumi-Sutherland, Alan Ruttenberg, Sirarat Sarntivijai, Ceri E. Van Slyke, Nicole A. Vasilevsky, Melissa A. Haendel, Judith A. Blake, Christopher J. Mungall

**Affiliations:** Department of Neurology, University at Buffalo School of Medicine and Biomedical Sciences, Buffalo, NY 14203 USA; European Molecular Biology Laboratory, European Bioinformatics Institute, Hinxton, Cambridge, CB10 1SD UK; ZFIN, the Zebrafish Model Organism Database, 5291 University of Oregon, Eugene, OR 97403 USA; Ontology Development Group, Library, Oregon Health and Science University, Portland, Oregon 97239 USA; Department of Biology, University of South Dakota, Vermillion, SD 57069 USA; National Evolutionary Synthesis Center, Durham, NC 27705 USA; Southwestern Medical Center, University of Texas, Dallas, TX 75235 USA; Unit for Laboratory Animal Medicine, University of Michigan Medical School, Ann Arbor, MI 48109 USA; Oral Diagnostics Sciences, University at Buffalo School of Dental Medicine, Buffalo, NY 14210 USA; The Jackson Laboratory, Bar Harbor, ME 04609 USA; Genomics Division, Lawrence Berkeley National Laboratory, Berkeley, CA 94720 USA

## Abstract

**Background:**

The Cell Ontology (CL) is an OBO Foundry candidate ontology covering the domain of canonical, natural biological cell types. Since its inception in 2005, the CL has undergone multiple rounds of revision and expansion, most notably in its representation of hematopoietic cells. For in vivo cells, the CL focuses on vertebrates but provides general classes that can be used for other metazoans, which can be subtyped in species-specific ontologies.

**Construction and content:**

Recent work on the CL has focused on extending the representation of various cell types, and developing new modules in the CL itself, and in related ontologies in coordination with the CL. For example, the Kidney and Urinary Pathway Ontology was used as a template to populate the CL with additional cell types. In addition, subtypes of the class ‘cell in vitro’ have received improved definitions and labels to provide for modularity with the representation of cells in the Cell Line Ontology and Reagent Ontology. Recent changes in the ontology development methodology for CL include a switch from OBO to OWL for the primary encoding of the ontology, and an increasing reliance on logical definitions for improved reasoning.

**Utility and discussion:**

The CL is now mandated as a metadata standard for large functional genomics and transcriptomics projects, and is used extensively for annotation, querying, and analyses of cell type specific data in sequencing consortia such as FANTOM5 and ENCODE, as well as for the NIAID ImmPort database and the Cell Image Library. The CL is also a vital component used in the modular construction of other biomedical ontologies—for example, the Gene Ontology and the cross-species anatomy ontology, Uberon, use CL to support the consistent representation of cell types across different levels of anatomical granularity, such as tissues and organs.

**Conclusions:**

The ongoing improvements to the CL make it a valuable resource to both the OBO Foundry community and the wider scientific community, and we continue to experience increased interest in the CL both among developers and within the user community.

## Background

The Cell Ontology (CL) was initially developed in 2004 with the goal of representing knowledge about in vivo and in vitro cell types [1]. Cells are a fundamental unit of biology, and most other entities in biology have direct relationships to identifiable cell types, for example particular proteins being produced by unique cell types, tissues and organs containing specific combinations of cell types, or biological processes being dependent on particular cell types. Cells therefore are an obvious set of entities to represent ontologically, and provide a useful pole for organizing and driving data acquisition and analysis in biology.

The content in the CL is populated via gradual and *en masse* class additions, most notably through several rounds of improvements to representation of hematopoietic cells in the ontology [2–4]. Originally, the CL was designed to include cell types from all major model organisms including both plants and animals [1]. However, as a result of community interest and severe resource limitations, continuing development of the CL currently focuses primarily on vertebrate cell types. The CL provides general classes that can be used for other metazoans (muscle cell, neuron), and the ontology can be extended in species-specific ontologies.

The CL is built according to the principles established by the OBO Foundry [5] and is the designated candidate ontology for metazoan cell types within the Foundry. The domain and content of CL is intended to be orthogonal to other Foundry ontologies to allow for the construction of compositional classes via logical definitions, as exemplified by the Gene Ontology (GO) [3, 6–8].

Work on the CL over the past several years has resulted in many improvements in the ontology’s structure and content. As described below, cooperation among a number of working groups has resulted in a modular approach to improving the CL, and continued enhancement of logical definitions in the CL have increased its integration and interoperability with other ontologies as well as enhancing its utility for data analysis.

## Construction and content

### Editorial management of the CL

The CL is maintained primarily by a small group of editors (ADD, YB, MH, DOS, CVS, NV, CJM), working in conjunction with interested parties from the ontology community. The editors use biweekly teleconferences to discuss significant issues related to CL ontology development. Because the CL has not been directly funded in recent years, most efforts are contributed as part of other projects and reflect the cooperative efforts of ontology developers and users based in different communities, such as the Gene Ontology Consortium [8, 9], the Immunology Database and Analysis Portal (ImmPort) [10], the Human Immunology Project Consortium (HIPC) [11], the Phenoscape project [12, 13], the Monarch Initiative [14], and model organism databases such as the Zebrafish Model Organism database (ZFIN) [15] and Mouse Genome Informatics (MGI) [16]. Consequently term creation occurs at an uneven pace, based on requests and editor availability. Over the past few years, we have received approximately 3–5 term requests per month. Most requests are accommodated in 1–3 months. The CL is released on an ad hoc basis, with new releases 5–6 times per year.

We welcome involvement of the community on particular domain specific developments, as has been done with kidney cell types (see below) and with immune cell types through our continuing collaboration with HIPC. Collaboration with the larger biological and biomedical community occurs both through our issue tracker and through direct contacts with any of the editors.

### Cell types in CL

As of June 2016, The CL contains approximately 2,200 classes, compared with 1534 at the time of our last report [3].The relative distribution of number of cell types among categories remains relatively constant, with one of the most well-represented being the hematopoietic cell branch, as described in [2–4], currently totaling 575 classes. Although the size of this branch has remained relatively constant, the content is continually refined and improved. For example, many of the original hematopoietic cell definitions are being reviewed and generalized to be applicable beyond mouse and human.

One area of expansion has been kidney cell subtypes, resulting from collaboration with the Kidney and Urinary Pathway Ontology (KUPO) project [17] as well as the Gene Ontology [18]. This has resulted in the addition of 125 new classes to represent kidney cell subtypes.

Over 400 cell types were added by generalizing human-specific classes from the Foundation Model of Anatomy (FMA) [19, 20]—many of these were compositional classes that we enhanced by adding both textual definitions and logical definitions connecting to Uberon. An example is ‘epithelial cell of thyroid gland’ (CL:0002257, FMA:0002257), logically defined as ‘endo-epithelial cell’ and (*part of* some thymus) [20].

### New and revised skeletal cell types

Work on the Vertebrate Skeletal Anatomy Ontology (VSAO), a unified ontology for the representation of skeletal cells, tissues, biological processes, organs, and subdivisions of the skeletal system [21], resulted in modifications to 13 existing cell types in the CL to ensure that the classes applied across vertebrates, and the addition of 18 new cell types. New relationships between cell types and skeletal tissues were also added, in addition to developmental relationships between skeletal cell types. These improvements enable broader queries on skeletal diversity across different biological scales. Improvements in the representation of skeletal tissues, organs, and subdivisions of the skeletal system have since been incorporated from VSAO into the Uberon multi-species anatomy ontology [22], and the logical definitions of associated cells to refer to the Uberon classes.

### Extending the CL to encompass vertebrate diversity

An ongoing challenge in developing the CL is to increase the number and granularity of cell types represented for well-studied species such as mouse and human, while providing high level classes needed for the representation of cell types in non-mammalian vertebrates. To ensure that CL classes are applicable to non-mammalian vertebrates two courses of action have been necessary: 1) add non-mammalian classes to the CL; 2) ensure that general cell type definitions do not unintentionally exclude certain organisms. Examples of non-mammalian cell types that have been recently added to the CL include the pigmented cells ‘iridoblast’ (CL:0005001) and ‘xanthoblast’ (CL:0005002) [23], and the ‘Kolmer-Agduhr neuron’ (CL:0005007) [24]. Ensuring that classes are applicable across species is a multifaceted problem and includes optimizing of cell type definitions, as well as (ideally) crafting class hierarchies that incorporate non-mammalian cell types from inception. Cell type definitions can unintentionally exclude non-mammalian vertebrates by including mammalian specific anatomical structures or by including species-specific proteins in the logical definition. At the same time, highly specified cell types for particular taxa are needed to enable querying of complex data using the CL. By adding less specific intermediate classes with inclusive definitions, such as multi-ciliated epithelial cell (CL:0005012), the CL can be used by a wide variety of model organism databases and evolutionary biologists for data annotation, while serving the needs of sophisticated bioinformatics analyses focused on cell types of medical interest.

### Improved delineation of content and coordination with other ontologies

**Fig. 1:**
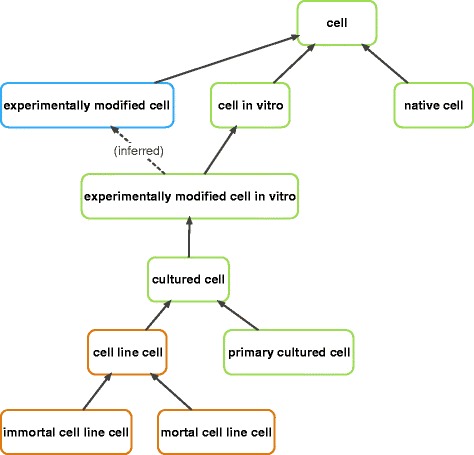
High level cell types in CL and related ontologies. The hierarchy of high-level cell types is shown. CL nodes: green, ReO: blue, CLO: orange

The primary focus of CL is to describe in vivo cell types [3], and while the priority of CL curators has been on in vivo cell types over the past few years, the ontology does in fact include a branch for in vitro cells. In order to clarify the representation of the domain of all cell types, representatives of the CL, Cell Line Ontology (CLO) [25], Reagent Ontology (ReO) [26], the Gene Ontology [9], and Ontology for Biomedical Investigations (OBI) [27], have agreed that the root class ‘cell’ (CL:0000000) in CL should be regarded as the root of all cell type classes in OBO Foundry ontologies (Fig. 1), and is equivalent to the GO class ‘cell’ (GO:0005623). As a result, changes were made to the upper level classes, to allow for a modular approach that represents in vivo and *ex vivo* cells types more accurately. Two of the children of the root class ‘cell’ are ‘cell in vitro’ (CL:0001034), and ‘native cell’ (CL:0000003) (which was formerly known as ‘cell in vivo’). The definition for ‘native cell’ reads as follows,

A cell that is found in a natural setting, which includes multicellular organism cells 'in vivo' (i.e. part of an organism), and unicellular organisms 'in environment' (i.e. part of a natural environment).

This definition reflects the fact that while cells of multicellular organisms are naturally considered ‘in vivo’ in their native state, single celled organisms often inhabit environments that are not part of another organism, and thus are not “in vivo” in that sense. The naturally occurring in vivo cell types of multicellular organisms are therefore properly considered subtypes of ‘native cell’.

Another agreed upon change in CL is that the classes ‘cell line cell’, ‘immortal cell line cell’, and ‘mortal cell line cell’ were deprecated (i.e., made obsolete) in CL and replaced with equivalent classes from CLO (see discussion below and Sarntivijai et al. [25] for additional details). As CLO specifically represents cell line cells, it seemed appropriate for CLO to contain its own root class and high-level cell type classes, and for the CLO developers to assume editorial control for these classes. Where needed, these three CLO classes were imported into CL using the MIREOT method [28, 29] to support existing annotations to these classes, and users of these classes, primarily MGI [16], were informed well in advance of these changes.

Similarly, ReO [26] contains the class ‘experimentally modified cell’ (Fig. 1) and a variety of related classes such as ‘genetically modified cell’ and ‘experimentally modified multicellular organism cell in vivo’. These cell type classes most commonly denote reagents of some type and fall outside of the domain of the CL proper, and clearly are within the domain of ReO.

Plant cell types and insect cell types are now handled independently of the CL as separate modules. The Plant Ontology (PO) has recently undergone new developments and the PO team has taken responsibility for curation of all plant cell type classes [30]. Consequently, all plant cell type classes in CL have been made obsolete. These plant cell types classes in the CL were already duplicates of existing PO classes, and were thus redundant and confusing to users. PO cell type classes may be imported into an extended version of CL as an OWL import in the future, retaining their PO IDs. [31]. A similar process is already used to create a pan-metazoan version of CL as part of the Uberon release process [32]; this will be extended to include *Viridiplantae*.

While the CL continues to represent a number of high level insect cell types, the Drosophila Anatomy Ontology (FBbt) contains cell types for many insect cell types not represented in CL, particularly insect neurons [33–35]. Similarly, the Zebrafish Anatomy Ontology (ZFA) also contains neuron types not represented in CL [36]. Going forward, the general approach is that non-mammalian species-specific cell types will be represented as *is_a* children of the appropriate CL parent in the species-specific anatomy ontology when such an ontology exists. The CL will continue to maintain general cell types for representation of non-mammalian cells where no separate resource or ontology exists and will remain the principal ontology for the representation of mammalian cell types.

As described above, the root class ‘cell’ (CL:0000000) in CL is declared to be logically equivalent to the GO class ‘cell’ (GO:0005623), within the Cell Ontology. While this arrangement mostly works for practical use of the CL, a long class proposal has been to deprecate ‘cell’ (CL:0000000) and simply make the GO class ‘cell’ (GO:0005623) to be the root of the Cell Ontology. However, there are still some minor differences in the way the two classes are defined, and questions about whether the Gene Ontology with its orientation to describing ‘normal’ or physiological biology should provide the CL root node ‘cell’, whose subtypes include tumor cell types, cell line cell types, and other experimentally modified cell types. This issue awaits additional discussion with the Gene Ontology Consortium and other interested parties.

### Natural Language and Logical Definitions in CL

The proportion of classes with natural language definitions has remained relatively constant, with a coverage of 82 % in both 2011 and the present. We still aim to boost this proportion to have 100 % coverage. The last five years have seen general improvements in logical axiomatization—in 2011 we reported the number of classes with defining equivalence axioms (logical definitions) to be 340, this number has increased to 1534, added through both manual and automated methods [3, 20].

**Fig. 2:**
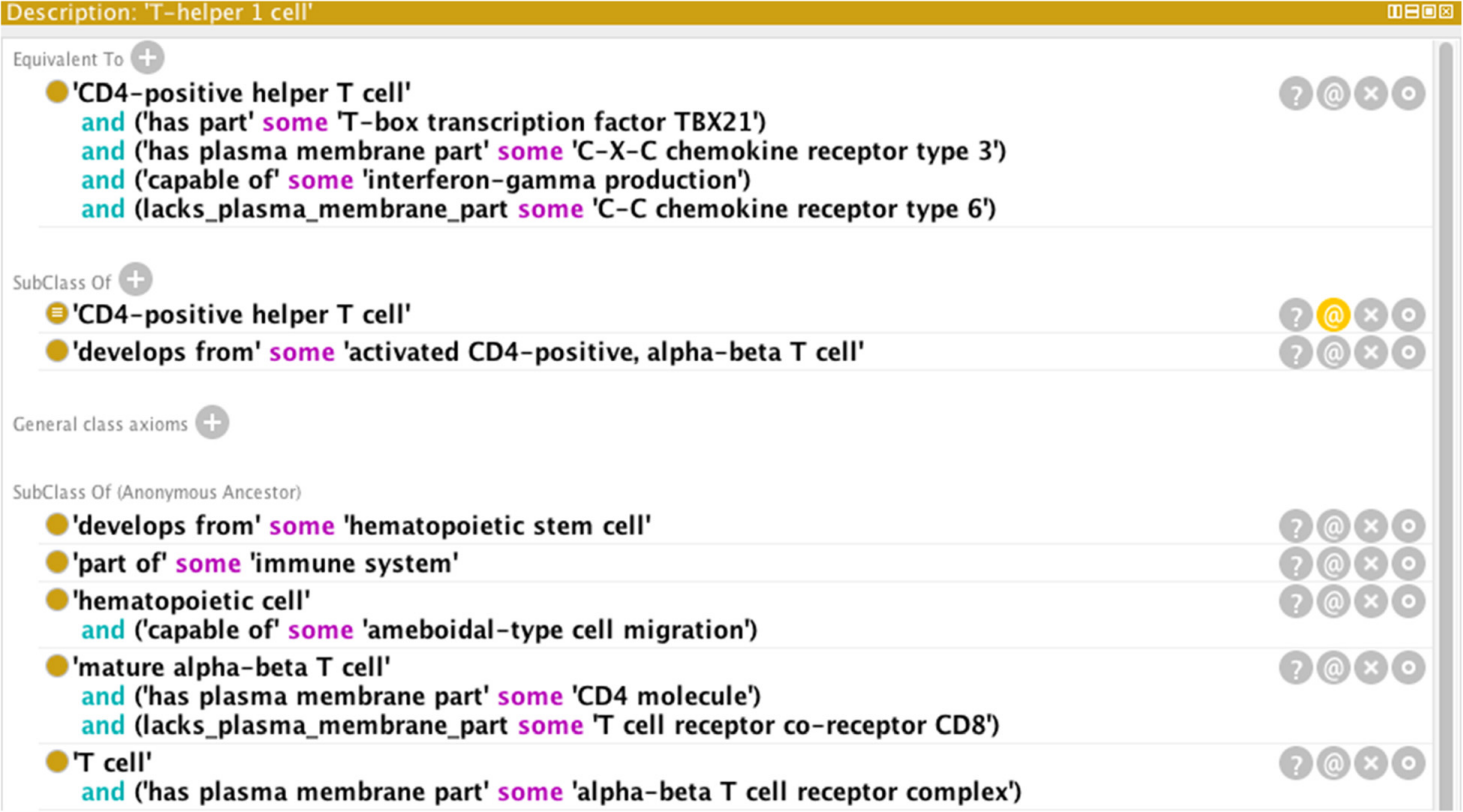
Logical definition for ‘T-helper 1 cell’ (CL:0000545). The logical definition for the cell type ‘T-helper 1 cell’ as presented by the Protégé ontology editor. The logical definition uses imported classes the Protein Ontology (‘T-box transcription factor TBX21’, PR:000001835; ‘C-X-C chemokine receptor type 3’, PR:000001207; and ‘C-C chemokine receptor type 6’, PR:000001202) and the Gene Ontology (‘interferon-gamma production’, GO:0032609). Note some anonymous ancestor classes are not shown due to space considerations

The set of ontologies imported into the CL to provide logical definitions remains constant, and consists of Uberon [22, 37], Protein Ontology (PRO) [38], GO [9], the Chemical Entities of Biological Interest (ChEBI) ontology [39], and the Phenotypic And Trait Ontology (PATO) [40]. Some classes make use of a variety of classes in the same axiom, such as ‘T-helper 1 cell’, which includes a mix of relations to both PRO classes and GO classes (Fig. 2).

### Improvements to nervous system cell types

In order to improve the representation of neurons and related cell types, we adopted the relations and methods originally developed from the Drosophila Anatomy Ontology [34, 35]. These include *synapsed_to* and *has_synaptic_terminal_in*, used to capture connectivity of neurons to each other and larger anatomical structures. We aim to coalesce with other neuron-specific vocabularies and ontologies, in particularly those that were part of the Neuroscience Information Framework (NIF) Standard suite of ontologies [41]. The analogous task has already been performed for neuron parts [42], and the gross neuroanatomical structure subset of NIFSTD has been incorporated into Uberon. As an initial task, we have aligned the contents of NIF-Cell with the CL by matching up identical or similar classes in the two hierarchies to identify gaps in both ontologies and differences in the ontologies’ structures. We will then define standard patterns for neuronal cell types, and import missing neuron cell types from NIF. In order to synchronize with the corresponding Neurolex wiki system, we have developed an approach for translating the Neurolex semantic wiki into OWL [43].

### Recent improvements in CL development methodology

The CL was originally developed using the OBO-Format and the OBO-Edit ontology editor [1, 44], without any automated quality control, release pipeline or automated procedures for building the ontology. We previously reported on improvements to this methodology, specifically leveraging the OWL2 ontology language [45] and associated tooling such as OWL reasoners, and the Protégé 5.x editor [20, 46].

We have made further changes and improvements to the ontology engineering framework we use. Previously, the editors’ version (source code) for the ontology was in OBO-Format, necessitating a conversion to OWL step prior to reasoning and debugging in Protégé. We have since switched the editors’ version to be in OWL, simplifying the procedure for working with the OWL stack of tools (note that we still produce editions in OBO-Format along with every release, as many bioinformatics tools still rely on this format). This switch also gives us greater flexibility for expressing concepts using the richer constructs available in the OWL language.

We have also implemented a TermGenie [47] instance, available at cl.termgenie.org. This provides a wider community of users a web frontend for instant provisioning of new classes, either conforming to pre-defined templates (i.e. design patterns), or templateless free-form additions. Currently the only design pattern implemented is a simple ‘part-whole’ template for the addition of classes like ‘epithelial cell of forearm’. One of the main users of the TermGenie instance has been the curators of the ENCODE project (see below).

We make use of the Jenkins Continuous Integration system, as developed and implemented by the Gene Ontology Consortium, for quality control and validation [8, 48]. This system alerts the editorial team if changes are made that inadvertently introduce logical, terminological, or structural errors into the ontology (for example, a cell that is located in two disconnected locations, or two cell classes that share the same name). We are in the process of switching to Travis-CI as this provides more direct integration with the GitHub system, where we manage the ontology. This system is also used to generate releases, creating a package of ontology files in OWL2 and OBO formats that are pre-reasoned and in some cases simplified for legacy use for systems that do not support logical definitions (See Table 1 for listing of available CL files).

**Table 1:** CL Ontology Files

File	pre-reasoned	with external ontology classes	PURL
cl-edit.owl	no	yes	N/A
cl.owl	yes	yes	http://purl.obolibrary.org/obo/cl.owl
cl.obo	yes	yes	http://purl.obolibrary.org/obo/cl.obo
cl-basic.owl	yes	no	http://purl.obolibrary.org/obo/cl-basic.owl
cl-basic.obo	yes	no	http://purl.obolibrary.org/obo/cl-basic.obo

In the time since we last published on CL, we have migrated the source repository we use to manage the ontology on two occasions. We originally migrated from SourceForge to GoogleCode; some time later, Google announced the retirement of GoogleCode, so we then followed many other ontologies and migrated to GitHub, where the source now resides [49]. Note however that most users of the CL do not interact with GitHub directly, and retrieve the ontology from the URLs provided in Table 1. Class requests and other inquiries for the ontology developers should be made through the CL issue tracker [50]. We have deprecated the older issue trackers on SourceForge and GoogleCode, and we migrated the tickets on these systems to GitHub.

While this migration process caused some disruption, this is compensated by efficiencies afforded by the GitHub system—for example, the ability to link edits on the ontology to tickets. The GitHub release mechanism also works well for ontology releases. One feature we hope to deploy this year is the ability to move to a GitHub-flow style of development, allowing external editors the ability to make ‘pull requests’ on the ontology, with complete validation being performed by Travis.

## Utility and discussion

### Use of CL classes in development of other ontologies

Cells are central to understanding biology from the molecular to the organismal level, and the CL is increasingly useful as a tool for representing and organizing cell types and data related to cell types in a variety of projects. As the designated ontology for the representation of cells in the OBO Foundry, the CL is used in a number of ontologies for the development of compositional classes via logical definitions. Gene Ontology developers have long employed the principle of “cross-product” class development, in which two classes from different ontologies are combined to make a more expressive “pre-composed” (or “compositional”) ontology class [6–8]. The class ‘neuron differentiation’ (GO:0030182), for instance has the logical axiom 'cell differentiation' and (*results in acquisition of features of* some ‘neuron’), where ‘neuron’ is a CL class. As GO developers continue to implement logical definitions for cross-product classes, they have increasingly needed new cell types in CL for use in these logical definitions. In order to facilitate this process, GO ontology developers have been trained in CL ontology editing as well and are now making direct contributions to the CL. An extended version of the GO that includes a subset of the CL together with linking axioms is available [51].

Development of the Cell Line Ontology (CLO) has referenced CL cell types and the hierarchy of the CL [25]. In CLO, all cell line cells are under the CLO class ‘cell line cell’, which is a child of CL ‘cultured cell’. Initially, CLO listed over 30,000 cell line cells immediately under the parent class ‘cell line cell’. To better identify the relations among different cell line cells, CLO generated many intermediate cell line cell classes (e.g., ‘immortal epidermal cell line cell’ and ‘immortal keratinocyte cell line cell’) based on a basic relation design that a CLO ‘cell line cell’ is derived from a CL ‘cell’, for example, CLO ‘immortal epidermal cell line cell’ *derives_from* some ‘epidermal cell’, and CLO ‘immortal keratinocyte cell line cell’ *derives_from* some ‘keratinocyte’. In CL, the class ‘epidermal cell’ is a parent of ‘keratinocyte’. Based on this CL hierarchical definition, CLO automatically includes a logical definition that ‘immortal epidermal cell line cell’ is a parent of ‘immortal keratinocyte cell line cell’. In total over 130 CL classes were imported to CLO with the hierarchy of the CL informing CLO structure. These newly generated CL-matched CLO classes were then used as parent classes for the over 30,000 cell line cells in CLO to layout an improved hierarchy of the cell line cells [25].

Interactions between different ontologies in the scope of biological cells can become complicated as we implement a thorough and precise representation of knowledge in this domain. As described above, CLO’s ‘cell line cell’ is a subclass of CL’s ‘experimentally modified cell in vitro’ where ‘experimentally modified cell in vitro’ is inferred as a subclass of ReO’s ‘experimentally modified cell’. The correct relationships among these related classes are only seen when all have been loaded into Protégé and a reasoner has been run. This degree of interrelatedness and complexity is becoming more common in bio-ontology practice, and demonstrates the needs for effective communication within the community. Being the center of interactions in situation like this, the CL has acted as the facilitating moderator of this kind of communication.

CL development allows for modular development of species-specific extensions. These extensions enable the creation of very granular cell types defined in ways that are unique to a particular species or limited to a subset of species. However, many cell types can be generically defined across species, and the CL provides the appropriate OBO ontology for their representation. In order to allow for comparison and integration of cell type specific data between species, species-specific cell types should always be subtypes of generic CL cell types. While development of modular extensions to CL is encouraged, the well-developed hierarchy of classes in the CL provides a valuable resource for data annotators working in species who do not have time or resources to develop CL extensions.

As examples of this methodology, developers of species-specific anatomy ontologies such as the Zebrafish Anatomical Ontology (ZFA) [36] and the Xenopus Anatomy Ontology (XAO) [52] have extended the CL by incorporating species-specific cell classes as *is_a* children of CL classes in their ontologies. This strategy allows ontologists to make species-specific classes that are *is_a* children of the appropriate CL class for use in data annotation at model organism databases. The integration of the CL with the species-specific ontologies also allows the CL classes to be used in phenotype and expression annotations at ZFIN [15] and expression annotations at Xenbase [53].

As ontologies such as the Infectious Disease Ontology (IDO) [54] or the Neurological Disease Ontology [55] are developed, CL classes are being used to represent information such as viral tropism or neurons affected in Parkinson’s disease. As with the GO, there is communication between developers of related biomedical ontologies that contribute to the development of both. The CL is also a component of the Experimental Factor Ontology (EFO), used to provide descriptions of experimental variables in databases at the European Bioinformatics Institute [56].

The CL is also being used far more extensively in the GO, in particular the GO has added a way to provide additional cellular context to gene associations using a mechanism called “annotation extensions” [57]. These cross-ontology linkages are used by a number of model organism databases in GO annotation and visible in AmiGO—for example, the page for ‘neuron’ includes GO annotations for neuronal parts [58].

### Use of the CL as Metadata in ENCODE and FANTOM5 Projects

Two major projects studying gene expression have utilized the CL as part of their data analysis pipelines. The Encyclopedia of DNA Elements (ENCODE) Consortium, which is funded and organized by National Human Genome Research Institute (NHGRI), aims to discover and define the functional elements encoded in the human genome [59]. ENCODE investigators are utilizing a prioritized set of various cell types to complete annotations about genes and their RNA transcripts and transcriptional regulatory regions and have developed data standards that utilizes the CL, among other ontologies, to describe the metadata for cell types used and experimental assays [60, 61].

The value of the CL for data integration and analyses was adeptly demonstrated in a recent series of notable papers from the FANTOM5 Consortium, which relied in part on the CL for large-scale data analyses of transcriptional start sites [62], enhancers [63], and waves of transcription in differentiating cell types [64]. The FANTOM5 Consortium utilized the CL as a component of the FANTOM Sample Ontology, in combination with Uberon, the Disease Ontology and the EFO to identify cellular, tissue, disease sources and experimental modifications for the samples used in transcriptional analyses [65]. By relying on the ontological hierarchy provided by the CL, the FANTOM5 Consortium was able to classify transcription patterns associated with individual cell types, groupings of related cell types, and cell lineages during differentiation [62, 64].

### Use of the CL in other non-ontology projects

The CL is being used as metadata in a variety of non-ontology projects, such as The Cell: An Image Library [66], CELLPEDIA [67], Phenoscape [13], LINCS [68], the Human Immunology Project Consortium (HIPC) [11], and ImmPort [10]. The HIPC and ImmPort projects are National Institute of Allergy and Infectious Diseases (NIAID) sponsored programs to collect and organize data from immunology experiments performed by NIAID supported investigators in order to facilitate secondary usage [10]. In support of these projects, the CL is being used both as a controlled vocabulary of cell types for use as metadata, and as part of an analytical pipeline for analyzing high-dimensional flow cytometry and mass cytometry data (e.g. CyTOF) [69] submitted to the ImmPort data repository. Developers of the CL have already incorporated novel B cell types discovered via high-dimensional flow cytometry [70], such as ‘IgG-positive double negative memory B cell’ (CL:0002103) and ‘IgD-negative CD38-positive IgG memory B cell’ (CL:0002107). The CyTOF method is yielding information about even more granular cell types [71]. In order to facilitate the analysis of data generated in high-dimensional flow cytometry or CyTOF, the flowCL software package matches cell populations identified via automated gating algorithms against existing cell types in the CL based on their combinations of markers, or immunophenotypes [72, 73].

## Conclusion

Through cooperative efforts between the Cell Ontology editors and various stakeholders, ongoing development of the CL has ensured that it continues to be a valuable resource for users and developers of related ontologies. Use of the CL by a broad range of third party efforts, including the high visibility ENCODE and FANTOM5 projects, as a source of metadata and for data integration and analysis shows the value of the CL to the wider scientific community. As big data collection and analysis continues to grow in importance as a source of biological discovery, we expect the CL will be of key utility in organizing and understanding these data. We invite community feedback and participation to continue the improvements to the CL.

## Availability and requirements

Like all OBO library ontologies, the CL is available from a standard purl [http://purl.obolibrary.org/obo/cl.owl]. The main URL for the project

[http://cellontology.org/] and links to various browsers are available from the main OBO Library page for CL [http://obofoundry.org/ontology/cl.html].
